# The Effect of Education Model in Physical Education on Student Learning Behavior

**DOI:** 10.3389/fpsyg.2022.944507

**Published:** 2022-07-08

**Authors:** Yongchao Chu, Chang Chen, Guoquan Wang, Fuzhi Su

**Affiliations:** ^1^Department of Sports Science and Physical Education, Guangzhou Xinhua University, Guangzhou, China; ^2^Department of Physical Education, Dongguan University of Technology, Dongguan, China

**Keywords:** education model, traditional physical education, learning behavior, *t*-test, control group

## Abstract

This research explores the effect of the sports education model implemented in physical education on college students' learning motivation and outcomes. The sports education model was compared with traditional physical education teaching as a control group. Participants were 60 college students in two classes. The ARCS (Attention, Relevance, Confidence, Satisfaction) Learning Motivation Scale, the Physical Education Affection Scale and a learning sheet were used for pre- and post-test comparison. Quantitative analysis was carried out on the post-test data using a dependent sample *t*-test and an independent sample *t*-test. The study found that: (1) the students in the sports education model group showed clear progress in learning motivation, affection, cognition and behavior, whereas the students in the traditional physical education group showed clear progress in cognition but no significant improvement in learning motivation, affection or behavior; (2) the sports education model group is clearly superior to the traditional physical education group in terms of learning motivation, affection, cognition, and behavior. This research shows that students are highly receptive to the sports education model, with a positive attitude and a high degree of motivation to learn to actively change their sports behavior. The sports education model brings several benefits: (1) it is an effective teaching method; (2) students' sense of responsibility, leadership and participation can be improved; (3) the preliminary homework and course structure descriptions take more time to compose, but can better guide students' motivation for learning physical education and can enhance teachers' professional growth.

## Introduction

Traditionally, physical education teaching has focused on the development of technology, but the application of technology was often neglected. This made students feel that physical education classes were monotonous, meaning that they lacked interest in learning, which affected their quality of learning. Traditional teaching often emphasizes description and demonstration of skills, with repeated drills to develop proficiency. Such teaching methods may not meet the needs of students and fully address the concept of physical literacy. Pedagogical thinking highlights that the focus of future development should be on the fit between teaching and competition or community activities. Therefore, some scholars have previously proposed that the extension of physical education classes is sports competition, as through sports competition students can enjoy the fun of sports, enhance their interpersonal communication skills, experience the potential value of diverse sports and cultivate autonomy. The American College of Sports Medicine (ACSM, 2013) promotes the sports education model, which emphasizes cultivation of “lifelong learners.” “Core literacy” refers to the knowledge, attitude, and ability that a person should possess in order to face future challenges and adapt to current life (Boung, 2010).

The curriculum structure in the field of fitness consists of two dimensions: the learning performance dimension and the behavior dimension, the latter being a combination of cognition, emotion and skills. The main focus is to support students to learn how to develop actions, such as making their own exercise plans and participating in outdoor activities, improving their motor skills and physical fitness, and demonstrating practical behaviors and attitudes, to achieve the goal of cultivating lifelong sports interest. The second focus is on learning content, which is divided into nine themes. Therefore, through the curriculum structure of learning performance and learning content, it is important to pay attention to the behavioral aspects of students and improve methods to create a good social environment. Teachers should focus on student-centered and situational learning perspectives, empowering students through team interaction to improve thinking and problem-solving skills in game situations. As traditional physical education focuses on the proficiency of movements and repeated operational skills, there is a substantial gap between the gained skills and knowledge and a real-world sports competition. The sports education model can be used to support students to learn how to participate in competitions, the meaning of fair competition and to develop lifelong learning skills in actual competitions. The focus is on the translation of movement into actual action and, most importantly, fostering positive social interactions. Through the sports education model, students can participate in sports, appreciate sports, and achieve the goal of competing in national sports (Hastie and Buchanan, 2000).

One way to achieve this is to allow students to take on more than one role, such as scorekeeper, referee, coach, etc. This allows them to achieve team goals in a democratic way and improves their development in all aspects and responsibilities. By taking on different roles in sports competitions, people can learn from a deeper, broader, and more positive educational sports experience. Students can learn responsibility, adaptability, enhanced skills and decision-making, with the acquired basic sports experience being more complete and enriched (Metzler, 2011). An important component of the sports education model is that it gives students a variety of learning opportunities, so that they can participate and enjoy the sense of achievement and joy of the game no matter what role they play, under the principle of fair competition. Students are supported to understand the concept of obedience and respect for others, so that those with poor skill levels can reduce their fear of sports and improve their learning motivation and outcomes. In teaching, motivation is one of the most important factors for good outcomes (Chen et al., 2022a,b) and the willingness of students to participate comes from their degree of motivation to learn (Hastie and Trost, 2002).

The ARCS motivation theory is divided into four elements, Attention, Relevance, Confidence and Satisfaction. It is a highly regarded teaching strategy that emphasizes that learning strategies should not only consider the characteristics and needs of students, but must also establish goals according to students' needs. Suitable teaching strategies can then be chosen for the set goals—and may be amended and evaluated at any time to improve teaching quality—so that students can generate and maintain motivation. However, in physical education, there are always students with lower or higher learning skills and motivation. Some students will start to hate or avoid physical education classes because they have no sense of achievement. Therefore, the curriculum design of sports education model needs to consider how to influence students with low levels of learning motivation. Siedentop (2002) highlighted that the sports education model has interesting and diverse activity designs, which can allow students to obtain better outcomes in terms of cognition, emotion and skills. Research into physical education teaching has found that students lacked interest in the physical education courses, motivation was low, and the learning outcomes were relatively low. Therefore, researchers must consider how to improve students' learning motivation and outcomes and how to put them into practical action. Sinelnikov and Hastie (2010) found that the teaching characteristics of the sports education model, including formal competition, group relationship and perceptual learning, are considerably attractive to students (more so than expected) and students continue to maintain a high level of motivation after the season is complete. Students' motivation to participate in sports and self-learning behavior can be effectively improved.

The present research is situated in the researcher's service school, where basketball is the main sport. In addition to regularly holding inter-class competitions, students also prepare for basketball competitions. Apart from long-term competitive players, most of the students are interested in basketball but only like to play by themselves and do not like to take classes. After considering many physical education models, the sports education model is most aligned with the researcher's own concept of teaching. The main purpose of this research is to explore the impact of the sports education model on learning motivation and outcomes in the teaching of physical education, specifically, basketball (Alexander and Luckman, 2001). The objectives are as follows:

1. To compare the influence of the sports education model and traditional physical education in basketball class on learning motivation.
2. To compare the influence of sports education model and traditional physical education model implemented in basketball class on learning outcomes.
3. To discuss perceptions and experiences of the implementation of the sports education model in basketball class.
4. To explore the problems and coping methods encountered in the implementation of sports education model in basketball teaching.

Participants are students who have been playing basketball for at least 3 years. The sports education model group (experimental group) comprises a total of 30 students (14 male, 16 female). The traditional physical education teaching group (control group) consists of 30 students (14 male and 16 female). Quantitative and qualitative methods are used in parallel, with learning motivation scales, emotion scales, study sheets, interview outlines, and teaching logs.

## Literature Review

### Education Model

The sports education model can take the form of a full teaching course or a teaching plan. It differs from the traditional teaching model as it contains an important process of role-playing, in which students can take on multiple roles such as captains, coaches, scorers, timekeepers, team members, etc. The focus is on giving low-skilled students a new opportunity to participate, to achieve a positive effect (Wallhead and Ntoumanis, 2004). Through this model, students can learn the full aspect of sports.

The curriculum objectives are the basis for curriculum value orientation. There are three levels: social culture, subject content, and individual development. Course objectives are also described via the learning outcomes. Curriculum objectives, teaching strategies and learning assessments can also be revised, thereby enhancing students' learning outcomes. The curriculum objectives of the sports education model are as follows (Chen et al., 2022c,d): (1) to develop students' cognition, emotion and skills; (2) to develop students' ability to solve problems and execute plans; (3) to enhance their appreciation of sports; (4) to foster good interpersonal relationships and team spirit. This shows that the sports education model not only focuses on sports skills, but also on more holistic aspects such as supporting students to become multi-faceted participants, to provide positive experiences and to learn how to share and communicate with others.

The basic concept of the sports education model comes from game theory, advocates the concept of national sports and encourages teaching plans to focus on various skills and physical fitness suitable for students of different levels. In a team environment of fair competition, students also need to learn how to make reasonable decisions in competitions (Huang et al., 2012, 2019). The curriculum is structured to motivate students to become skilled individuals who understand the rules of sport.

The sports education model has six main characteristics:

(1) Season: implementation follows the sports season which is a different model from traditional teaching. Only one sport is studied in a season. Usually, a sports season is about 4–6 weeks. Teachers can design courses according to the time of the sports season and follow the gradual mastery of sports skills, knowledge, tactics, and other aspects through different course units.

(2) Affiliation: teachers divide students into several groups through heterogeneous grouping, ensuring that the abilities of each group are as even as possible. The members of each group are assigned roles according to their own abilities, such as captain, referee, timekeeper, scorekeeper, etc. The main purpose is to foster a sense of teamwork and group cohesion, in order to increase students' self-concept, so that regardless of their ability, students can feel a sense of belonging in the group.

(3) Formal competition: the focus of model is that the formal competition is not a group practice competition based on traditional physical education teaching. Rather, the competition system is diverse, including round-robin competitions, confrontation competitions, etc. In the formal competition, students are encouraged to compare techniques, which can give them more incentive and stimulation.

(4) Final period: this is the climax of the model, usually the championship game. The purpose is to increase overall visibility and, through this competition system, students can be motivated to participate and enhance their sense of identity as a team. Sports skills are not the only teaching objectives and more attention should be paid to improving students' interest in participating in activities in order to achieve their goals.

(5) Record keeping: the entire sports season is like a learning archive, which records the various performances of the students in the competition, in the role-play work, etc. and provides feedback to the students to make corrections (Ye et al., 2019, 2021; Fu et al., 2022). Teachers can also learn about students' learning status, so as to adjust the curriculum and improve teaching.

(6) Festivity: one of the learning goals is to let students understand the meaning of sports rituals. From the beginning of the sports season, the team establishes team names, team calls, flags and other designs, from the opening to the closing. The whole sports season should be full of celebration, so that students can feel joyful and enthusiastic to devote themselves to the course, and achieve the goal of participating in sports. This sense of festivity is lacking in traditional physical education (Perlman, 2010).

When the model is implemented, teachers make use of the modification of rules, changes in venues, changes in equipment, students' operation in team combinations, and role-playing, so as to allow each student to develop strategies, skills, learn cooperation, and experience success. Under the environment of fair competition, low-skilled students are encouraged to reduce their fear of sports, increase their motivation to participate, and all students learn to respect others (Chen et al., 2020, 2021). This approach aligns with curriculum policy in China, which encourages a focus on students at the center, with everyone having the opportunity to participate in physical activities.

### Learning Motivation

Learning motivation is an internal process that causes and sustains an individual's activity (behavior) toward a goal. Motivation has five properties: (1) it can explain behavioral goal orientation; (2) it can determine activity time; (3) it can determine reinforcement; (4) it can be an end or a means; (5) it never exists in a vacuum. Therefore, understanding how to improve students' learning motivation is very important for teachers, because it will affect students' learning behavior.

The ARCS model was intended to promote the teaching of courses in a way that maintains motivation during activities. Cheng and Yeh (2009) highlighted that the model is based on the theory of expected value. It follows the perspective of human motivation in educational psychology and can be effectively integrated and applied with other teaching models. The ARCS motivation theory can stimulate students' learning motivation in learning and improve their achievement level. When teaching courses attract students' interest, they improve concentration on learning. Teachers can make changes to materials, use different approaches such as videos or demonstrations, or provide students with more relevant life topics so that students can learn more meaningfully. The four elements of ARCS, which need to be used together, are as follows:

(1) Attention (A) refers to whether the course can arouse students' interest or curiosity, that is, whether students can feel that the course is interesting and worth paying attention to.

(2) Relevance (R) refers to the need for the teaching objectives to meet the needs of students when they are learning so that they understand that what they have learned is relevant to their lives.

(3) Confidence (C) affects level of effort and performance, and refers to the extent to which students recognize that they have the ability to successfully complete learning tasks after a period of effort. Teachers should consider the difficulty level when designing courses.

(4) Satisfaction (S) refers to an evaluation of students' learning results in teaching and learning, that is, the degree of satisfaction they feel both internally and externally. This is an important factor in making learning motivation sustainable.

The key to how to arouse and maintain learning motivation lies in how teachers use their professional ability and instructional design or methods. Teaching should follow the asymptotic approach as much as possible and use their skills to guide learning motivation, promote intrinsic motivation and reduce extrinsic motivation, so as to enable students to maintain motivation and achieve the goal. ARCS can effectively improve learning motivation and learning outcomes. Keller (2010) also highlighted that the ARCS motivation model is integrated into the teaching curriculum, which has obvious positive effects on teaching and learning. The ARCS motivation theory is the most used and discussed of many similar models and has shown clear results in physical education. Therefore, it is one of the more suitable research tools for the present study.

### Learning Effect

The learning effect, or learning outcomes, refers to the knowledge learned from the course and the ability to use the knowledge. Questionnaires, observations, records, and checklists can be used to evaluate the learning outcomes of students and to know how well the students are meeting the learning objectives. Improving learning can also improve teaching (Perlman, 2010). The learning effect can be assessed in three different domains. The cognitive domain assessment focuses on the learning of the mind. Emotional or affective assessment is more difficult than skills assessment and cognitive assessment, as it must be “transformed” into specific goals. In the past, only three dimensions of cognition, affection and skills were focused on in teaching. Behavioral assessment is a newly added dimension of learning performance. The main purpose is to hope that students can transform learning into actions and achieve the attitude and behavior of automatically and spontaneously formulating exercise plans and practices. This would mean that the goal of a lifelong movement has been reached (Perlman, 2010).

### Sports Education Model

The sports education model has nine major benefits for physical education (Boung, 2010):

(1) Modification and reduction of restrictions on competition rules: the rules of the game that were originally more complicated or require a higher level of skills become simple and easy to use, so that students with lower skills can also participate.

(2) Role-playing gives everyone the opportunity to participate: the role-playing component allows students to perform a variety of roles, such as referees, scorers, coaches, etc., in cooperation with each other. This allows them to develop the idea of the team being bigger than the individual, and problem-solving skills.

(3) Changes in the role of teachers: the role of teachers has changed from simply providing direct guidance. When students really have problems that cannot be solved by the team, they will guide students to solve problems, or will give idea back to the group to let students think about how to solve problems.

(4) It takes a long time to design the course: breaking away from traditional teaching, it takes several weeks to design a course based on the sports education model. It is not only a talk, but also pays more attention to group discussions and operations.

(5) Multiple evaluation methods: traditional methods of evaluation use an objective method, but the sports education model uses various evaluation tools such as subjective scales, objective scales, study sheets, competition performance, and teaching records. This also allows teachers to revise their own teaching curriculum design.

(6) Increased communication and interaction among peers: in most of the research literature, there is a significant improvement in the increase of interpersonal interaction, because students must communicate through group discussion to solve the tasks given by teachers in the course.

(7) Decentralization and autonomy of teaching power: teachers delegate part of the power of teaching to the team leader, so that the leader can develop leadership ability, division of labor, communication, and give students the ability of self-management. Teachers also help students to think and develop problem-solving skills through feedback to reduce conflict.

(8) Establishment of a reward system: the model adheres to the principle of everyone being rewarded. It is hoped that students can be encouraged after the end of the entire season, so that students do not feel that the course is over.

(9) Learn to appreciate sports competitions and sports rituals: by observing the performance, tactics and movements players can have the opportunity to use these skills again in subsequent games, and will not be confused about the rules on or off the court. The model uses a variety of learning methods, so that students have a range of goals, not only skills-based, but also in cognition, affection competition performance, sports behavior, and physical fitness. Research has shown that traditional physical education teaching has significantly higher proportions of dissatisfied students, but the autonomy and support of the sports education model can help with this by increasing a sense of team belonging (Boung, 2010; Perlman, 2010). The model also influences teachers' professional development; teachers unanimously believe that the sports education model is a good teaching method and that the sense of responsibility, leadership, and student participation have all been improved, although the courses take more time to compose.

## Research Methods

### Research Structure

**Figure 1:**
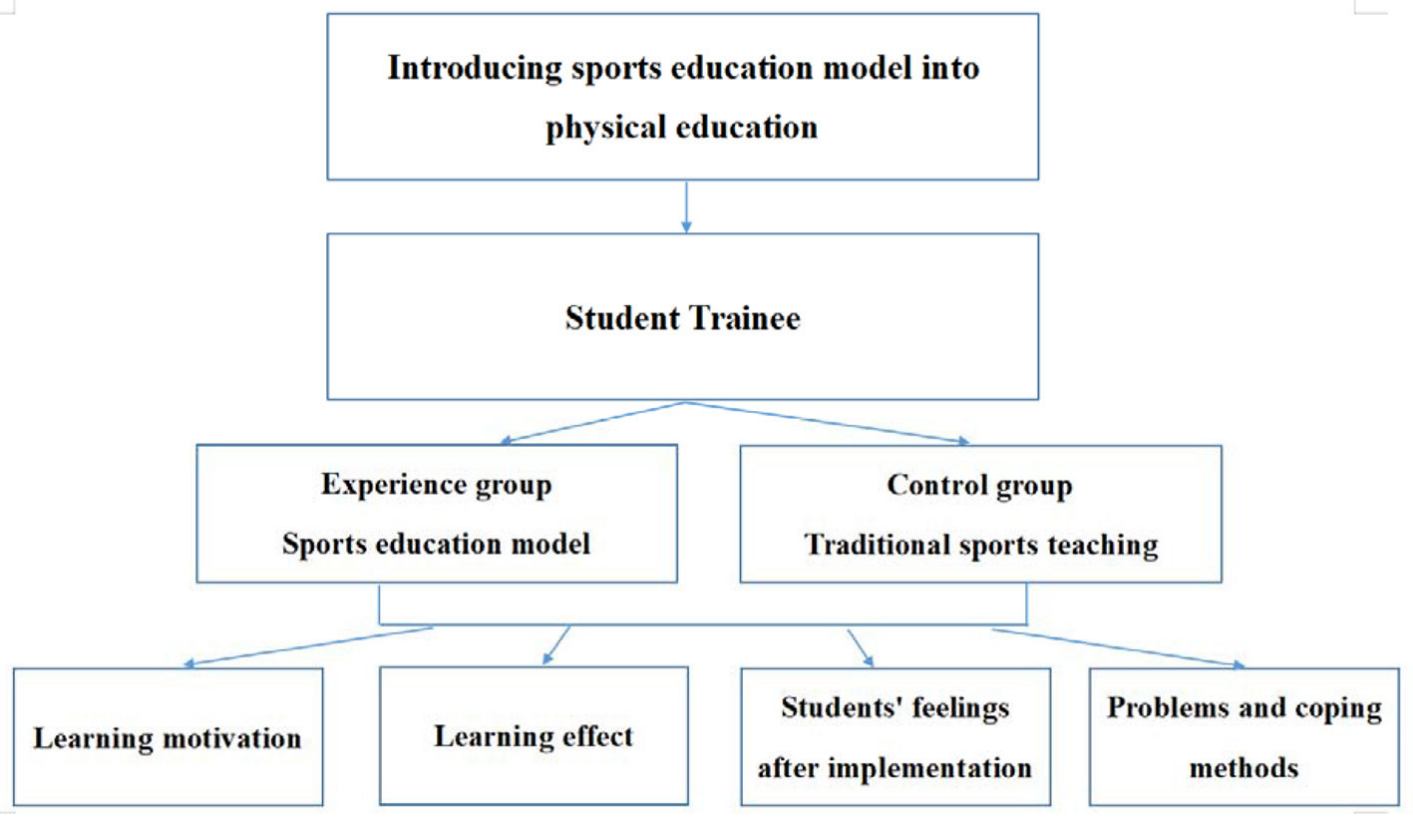
Research structure.

The present study designed 8-week, 16-session physical education activities and explored the learning motivation and learning outcomes of students during the implementation of the sports education model. The study also explored students' feelings after implementing the sports education model and the problems and coping methods encountered by teachers in implementing the model. Figure 1 shows the structure:

### Research Participants and Process

A total of 60 students in two classes were selected, an experimental group (14 boys and 16 girls) and a control group (14 boys and 16 girls). None of the students had received physical education via the sports education model.

The study adopted a quasi-experimental design. Each outcome measurement scale was produced before the experiment and was subjected to reliability and validity analyses. Scales were used pre- and a post-test. Suitable evaluation measures were identified from the physical education literature.

### Evaluation Tools

In order to understand the changes in learning outcomes and learning motivation after implementing the sports education model, this research chooses to use both qualitative and quantitative evaluation methods: (1) ARCS Learning Motivation Scale; (2) Physical Education Affective Scale; (3) Interview; (4) Study sheet; (5) Teaching checklist.

(1) ARCS Motivation Scale: Keller proposed that the ARCS model can stimulate learners' motivation to learn, and there are four factors: (1) Attention: whether the reference can arouse students' curiosity or interest; (2) Relevance: whether students can perceive that the course meets the target expectations or meets the needs of students, and allows them to understand that the knowledge they have learned is relevant to them; (3) Confidence: whether students can perceive that they can successfully complete the task after hard work; (4) Satisfaction: the intrinsic and extrinsic rewards that students receive in the learning process. The ARCS learning motivation theory scale used in this study was the Physical Education Learning Motivation Scale. Each of the four factor has four questions, assessed via a five-point Likert scale.

(2) Physical Education Affection Scale: the purpose of this study is to investigate whether students' affection has changed after the implementation of the sports education model. School sports implementation goals, and teaching goals in the field of health and physical education were used to prepare test questions. The scale showed good quality when tested on the three major qualities of reliability, validity and norm or standard required for scale preparation.

(3) Semi-structured interviews were used to understand the inner feelings of the interviewees.

(4) Study sheet: the study sheet was used to understand the students' thoughts and feelings on learning after the implementation of the sports education model, as well as their preference for various aspects of role-playing. Teachers can also reflect on the learning of students via the study sheet.

(5) Teaching behavior checklist: before the study, it was necessary to check whether the teaching behavior of physical education teachers conforms to the sports education model, so that the data and results obtained after the experiment are meaningful. Two physical education teachers with more than 10 years of teaching experience in the school were invited to serve as observers. The researcher first explained to the two observers the characteristics and inspection items of the pedagogical method of the observation and gave relevant information on the pedagogical method. The teaching reliability calculation formula used is the intrinsic observer reliability formula. The results of two observers' inspection of the same teaching are brought into the formula to calculate the internal reliability between observers. The teacher's teaching behavior check reached 91%, and the reliability of more than 0.80 was an acceptable standard. It was therefore shown that the sports education model can be taught reliably.

### Data Collection and Analysis

Quantitative data were analyzed through SPSS for Windows statistical software, with the statistical significance level of the statistical test was set as α = 0.05. Data processing used the following procedures:

Dependent sample *t*-test: the teaching groups' pre-test and post-test differences in terms of affection, learning motivation were assessed.

Independent sample *t*-test: the differences in post-teaching affection and learning effect between the two groups were tested.

Covariate analysis: if there is no significant difference in the pre-test via the independent sample *t*-test, the independent sample *t*-test will be carried out on the post-test scores.

Qualitative data were classified and coded according to the research questions. The data was made anonymous and numbered, before being transcribed and organized. Correlation between the data were explored and interpreted according to the research question. The qualitative data obtained from the research is triangulated with study sheets, interview records and teaching logs, and different statements of the same situation are compared and contrasted, so as to summarize the similarities and differences. Using different methods to collect data for triangulation and consulting experts and scholars can improve reliability and validity.

## Findings and Discussion

The research results discuss the influence of the sports education model and traditional physical education on basketball learning effects, the experiences of the sports education model, the problems encountered by teachers in the implementation and the coping methods.

### The Influence of the Sports Education Model and Traditional Physical Education on Basketball Learning Motivation

#### Results

We used the Physical Education Learning Motivation Scale to detect the differences between students before and after the implementation of the sports education model. As shown in Table 1, the pre-test average of the 30 students in the study was 60.87 ± 8.37; the post-test average was 64.17 ± 7.58 and the dependent sample *t*-test was −0.141, *p* < 0.05. This shows that the students participating in the sports education model group had a significant level of basketball learning motivation before and after the test, and the mean of the post-test is higher than the mean of the pre-test. Therefore, after the implementation of the sports education model, learning motivation improved significantly.

**Table 1:** Pre- and post-test *t*-tests of the learning motivation effect of the exercise education model.

**Evaluation**	**Stage**	**Number**	**Mean**	**Standard deviation**	***t* value**
Learning motivation	Pre	32	61.31	8.28	0.292
	Post	32	59.06	8.86	

Table 2 shows the summary of the learning motivation effect of traditional physical education. The average pre-test of the traditional physical education group was 61.31 ± 8.28, which shows that the group did not reach a significant level of basketball learning motivation before and after the test. The pre-test average was higher than the post-test average, which means that the traditional physical education group did not see a significant difference in basketball learning motivation after teaching.

**Table 2:** Pre- and post-test *t*-test of the learning motivation effect of traditional physical education.

**Evaluation**	**Stage**	**Number**	**Mean**	**Standard deviation**	***t* value**
Learning motivation	Pre	32	61.31	8.28	0.292
	Post	32	59.06	8.86	

Next, we compared the learning motivation of the sports education model and traditional physical education teaching. The sports education model group and the traditional physical education group were pre-tested on the effect of learning motivation and the results of the two groups of homogeneity tests through the independent sample *t*-test are shown in Table 3.

**Table 3:** Comparison of the two groups: pre-test learning motivation effect t-test.

		**Sports education model**	**Traditional physical education teaching**	
**Assessment item**	**Stage**	**(*N* = 30)**	**(*N* = 32)**	***t* value**
		Mean, standard deviation	Mean, standard deviation	
Learning motivation	Pre-test	60.87, 8.37	61.31, 8.28	0.884

*p < 0.05.

As shown in Table 3, the average pre-test score of learning motivation in the sports education model group was 60.87 ± 8.37; the average pre-test of learning motivation in the traditional physical education group was 61.31 ± 8.28 and the *t* value of 0.884 was not significant. The results show that the two groups of students are homogeneous, so the post-test scores can be used to conduct an independent sample *t*-test to analyse the differences in learning effects after teaching. These research results are shown in Table 4.

**Table 4:** Comparison of the two groups: post-test learning motivation effect *t*-test.

		**Sports education model**	**Traditional physical education teaching**	
**Assessment item**	**Stage**	**(*N* = 30)**	**(*N* = 32)**	***t* value**
		Mean, standard deviation	Mean, standard deviation	
Learning motivation	Post-test	64.17, 7.58	59.06, 8.86	−0.101*

*p < 0.05.

As shown in Table 4, the mean post-test of learning motivation in the sports education model is 64.17 ± 7.58; the mean post-test of learning motivation in the traditional physical education teaching is 59.06 ± 8.86, and the obtained *t* value is −0.101 < 0.05, which is significant. Therefore, according to the above results, there are significant differences in learning motivation between the sports education model group and the traditional physical education group.

#### Discussion

The results of this study show that there is a significant difference in the performance of learning motivation before and after the implementation of the sports education model. The sports education model adopts a seasonal approach which allows students to learn through competitions, thereby increasing their willingness to participate. The team group method can promote students to develop the ability to make decisions and take responsibility for learning, thereby improving their learning motivation. Perlman (2010) highlighted that the sports education model can significantly increase unmotivated students' interest in physical education and satisfaction in interacting with their peers. The sports education model can enhance students' motivation to study, give them a sense of achievement and satisfaction in sports, and cultivate the habit of exercising after school. Therefore, if the teaching method and design can be used properly, and the curriculum can be designed according to the needs of students, the physical education class will not be standardized but interesting. Role-playing in the sports education model is to give students learning goals so that they can enough to increase the motivation to learn. Clear goals can improve motor learning motivation.

The results of this study also show that there was no significant difference in traditional physical education before and after the test. Traditional physical education courses are usually teacher-led, via presentations, demonstrations, groupings, and standardized practice methods. The focus skill learning, and the repeated practice of single action skills makes students unable to enjoy games, fail to learn how to apply strategies in competitions, fails to stimulate motivation and make students lose sight of their real learning goals.

The sports education model group was significantly better than the traditional physical education group. The traditional physical education teaching mode is limited by the curriculum set by the school, so the sports must be changed frequently, which leads to difficulties in learning for students. However, it is found that the sports education model has a good effect on students' learning motivation, competition performance and strategy application. The sports education model can improve the motivation of students to participate in sports, and can more effectively induce the autonomous learning of group members and play a role in teamwork. Therefore, the sports education model is helpful to improve students' motivation in physical education.

### The Influence of the Sports Education Model and Traditional Physical Education on Basketball Learning Outcomes

#### Results

For the quantitative analysis, the homogeneity test was used first and the independent sample *t*-test was used to analyse whether there was any difference in the pre-test scores of the two groups of students. If the two groups are not significant, the post-test scores can be used independently. The qualitative aspect mainly discusses the difference between the cognitive learning effect and the behavioral learning effect between the sports education model and traditional physical education. The analysis of the affective effect of the sports education model (Table 5) showed that the mean of the pre-test of the 30 students in the study was 155.94 ± 16.78; the mean of the post-test was 173.57 ± 15.95, and the t value obtained by the dependent sample *t*-test was −0.435, *p* < 0.05. The students in the sports education model group saw a significant pre- and post-measurement effect on affective learning. The post-test mean was higher than the pre-test mean, therefore, after the implementation of the sports education model, there was a significant difference in the affective learning effect.

**Table 5:** Pre-test and post-test t-test of affective learning outcomes of sports education model.

**Evaluation**	**Stage**	**Number**	**Mean**	**Standard deviation**	***t* value**
Affection	Pre-test	30	155.94	16.78	−0.435*
	post-test	30	173.57	15.95	

As shown in Table 6, the pre-test mean of the affective effect of the traditional physical education group was 163.09 ± 20.24; the post-test mean was 164.78 ± 20.53, and the t value obtained by the dependent sample *t*-test was 0.742, *p* > 0.05. This indicates that the traditional physical education teaching group did not achieve significant results in the pre-test and post-test, and the post-test average was close to the pre-test average. Therefore, the traditional physical education group did not see a significant difference in the affective learning effect after teaching.

**Table 6:** Pre-test and post-test *t*-test of affection effect of traditional physical education teaching.

**Evaluation**	**Stage**	**Number**	**Mean**	**Standard deviation**	***t* value**
Affection	Pre	32	163.09	20.24	0.742
	Post	32	164.78	20.53	

To compare the affective effect of the sports education model and traditional physical education teaching, both groups were pre-tested on the effect of affective learning. The results of the two groups of homogeneity tests via the independent sample *t*-test are shown in Table 7.

**Table 7:** Homogeneity test of the affective effect pre-test comparing the two teaching groups.

		**Sports education model**	**Traditional physical education teaching**	
**Assessment item**	**Stage**	**(*****N*** **=** **30)**	**(*****N*** **=** **32)**	***t*** **value**
		Mean, standard deviation	mean, standard deviation	
Affection	Pre-test	155.94, 16.78	163.09, 20.24	0.446

Comparing the affective effect summary (Table 4) of the two groups, the average pre-test score of the sports education group was 155.94 ± 16.78, the traditional physical education group was 163.09 ± 20.24 and the *t* value was 0.446, which was not significant. The research results show that the two groups of students are homogeneous, so the post-test scores can be used to conduct independent samples *t*-test to analyse the difference in learning effect after teaching. Table 8 shows the results of the independent sample *t*-test of the teaching post-test.

**Table 8:** Comparing the two groups of teaching affection post-test learning effect *t*-test.

		**Sports education model**	**Traditional physical education teaching**	
**Assessment item**	**Stage**	**(*****N*** **=** **30)**	**(*****N*** **=** **32**)	***t*** **value**
		Mean, standard deviation	Mean, standard deviation	
Affection	Post-test	173.57, 15.95	164.78, 20.53	−0.416*

*p < 0.05.

As shown in Table 8, in terms of affective learning effect, the post-test mean of the sports education model was 173.57 ± 15.95, the post-test mean of traditional physical education was 164.78 ± 20.53 and the *t* value was −0.416, *p* < 0.05, which was significant. Therefore, there is a significant difference in affective learning effect between the sports education model group and the traditional physical education group.

Regarding cognitive learning, according to the interview data, before the implementation of the sports education model the majority of the students were not clear about the rules of basketball, the key actions and the signals of the referee. Some students did not understand the most basic rules, such as walking, double dribbling and one-ball counting. After the implementation of the sports education model, understanding of the rules, key actions, and referee signals all improved. Students gradually changed from passive participation to active participation. The implementation of the sports education model has therefore improved students' basketball cognition, and allowed them to gradually move from being confused to following the correct learning path. Everyone began to discuss the game, study movements, assist teammates and use the peer support to encourage everyone to learn and grow together. Similarly, after the implementation of traditional physical education teaching, most students made significant progress in their understanding of basketball rules, key actions, referee signals and other related information. When comparing the cognitive learning effect of sports education model and traditional physical education teaching, both groups saw obvious growth in cognition. However, after the implementation of the course, the students in the sports education model group showed obvious effects in cognitive knowledge, understanding, analysis and application; the traditional physical education group only made significant progress in cognitive knowledge and understanding.

Regarding behavioral learning, before the implementation of the sports education model, students were generally passively participating in, or rejecting, physical education classes. The unfocused teaching mode and the current education policy meant that students had a more negative attitude and did not want to try anything new. After the implementation of the sports education model, the teamwork and the division of roles helped students to actively participate in physical education classes. They felt that they had improved their understanding of basketball and were more interested in watching some basketball games, wanting to see what tactics were used and what decision the referees were making. Those who were assigned non-playing roles (e.g., recorder) felt that even though their basketball skills were poor, they had something to do without feeling isolated. After the implementation of the sports education model, students' learning of basketball became active, with group discussion and planning. They began to discuss in class how basketball should be practiced and where it should be strengthened. The introduction of the sports education model therefore brought a great change in students' behavior. In comparison, after the implementation of traditional physical education teaching, the students' behavior did not changed significantly and they still just follow the teachers' practice or returned to the previous learning method. Therefore, after the sports education model and traditional physical education model are implemented, the students in the sports education model group showed a greater and more obvious improvement in behavior, but there was no difference in the traditional physical education group. After implementation, students in the traditional physical education group may want to improve physical fitness, practice after class and actively learn about basketball-related information. Some students think that studying is more important. Therefore, it is reasonable that the students in the sports education model group showed significantly better behavioral learning effect than the students in the traditional physical education group.

#### Discussion

The results of this study show that there is a significant difference in affective learning before and after the implementation of the sports education model. The sports education model has an important process of role-playing, which hopes that students with low skill levels can also participate in sports in different team roles. According to the quantitative statistical analysis, there is no significant difference in the effect of traditional physical education before and after on affective learning. The reason is that the traditional physical education method focuses more on skill learning and achieves learning goals through practice. In other words, students are more passive in learning the teacher is at the center to explain and demonstrate movements, so the students do not learn how to think and use what they have learned. When comparing the affective learning effect between the sports education model and the traditional physical education model, the sports education model group was significantly better than the traditional group. This is because that the sports education model has relatively complete and rich features such as a team group, role division, competition season, award ceremony, etc., but traditional physical education focuses on standardized movement skills training so that students do not enjoy learning.

The students' cognition of basketball before implementation was quite unclear, but after implementation, they all improved and were able to better understand basketball rules, tactics, referee signals, and other related information. The sports education model emphasizes team learning, peer communication and interaction, and the establishment of a class atmosphere, while teachers change from leaders to guides. Students also need to start thinking about tactics and problem-solving strategies, and can understand their own roles and responsibilities. Therefore, the sports education model is helpful for students to improve cognitive learning. According to the qualitative data, the cognitive learning effect of traditional physical education also showed significant improved. The reason may be that the previous physical education methods paid more attention to the design of skills or school curriculum, resulting in the neglect of cognitive knowledge in teaching. In this study, the course time was increased because of the needs of the experiment. In long-term teaching, teachers and students were also given longer practice and teaching time, and a competition mode was added at the end, so that students could go through the competition. When comparing the cognitive learning effect of the sports education model and traditional physical education model, both groups made obvious progress after implementation compared with before implementation, so there is no special difference in cognitive learning effect. The only difference is in the system; the sports education model has a complete season while the traditional physical education model takes every lesson.

In terms of behavioral learning effects, students were relatively passive before implementation and if there was no test, they would not take the initiative to practice. The sports education model encourages the team relationship and peer support, and there are also clear roles and responsibilities, which in turn prompts students to want to know more about basketball-related information, and increases the number of after-school sports and practical actions for sports planning. The sports education model can help students to produce significant changes in behavior. In contrast, there is no obvious change in the behavioral learning effect of traditional physical education teaching. The teaching mode will cause students to lack thinking ability, application ability, and motivation. The behavioral changes of the students in the sports education model group are therefore significantly better than those in the traditional physical education group.

After the implementation of the sports education model, students developed good exercise habits. Most would exercise together after school or during holidays and learning motivation could be effectively improved. Most teachers agree to adopt a variety of teaching strategies, but in the field of education, most teachers still take the approach of the teacher as the center and adopt didactic methods that emphasize repeated practice. Sinelnikov and Hastie (2010) highlighted that the teaching characteristics of sports education model, such as team groups, competition seasons, sports seasons, etc., are more attractive to students and will leave them with a high degree of interest after the course is over. The team aspect of the sports education model allows students to have more space for discussion and thinking. Therefore, it is understandable that the behavioral effect of the sports education model is better than that of traditional physical education teaching.

### The Implementation of the Sports Education Model in Basketball Classes

The analysis of findings regarding the implementation of the sports education model can be summarized as follows:

(1) Students no longer occupy a single role in learning; everyone has the opportunity to take up important positions and everyone is indispensable. Students realize that even if their skills are poor, they can still take up other roles to help the team. There will always be students with better and less ability in a team. In previous courses, ability was poor and students were often afraid to participate, fearing that they will be a burden. The division of labor emphasized by the sports education model allows these low-skilled students to perform functions in other roles. Students then realize that the completion of a ball game is not only about the players on the field, but also needs the assistance of many people behind the scenes.

(2) The sports education model is more popular with students than traditional physical education teaching, and it is also more complete and richer, prompting students to fall in love with physical education classes. Most students think that learning the same project for a long time can make them more proficient in a certain sport but a small number of students think that the time is too long. In the past, physical education teaching made students think that physical education class is very boring and meaningless, resulting in them not wanting to participate in the course. Students feel that the physical education class is more exciting than the season part, there are more specific goals in learning and they have more motivation to work hard. Traditional physical education teaching neglected the “comparison of the season” aspect.

(3) The concept of the team group is very helpful for students with low motivation or skills. Teaching in a team group mode can transmit the course information more quickly, and influence the motivation of the people in the group through peer relationships. This allows students with low learning motivation to participate in exercises, competitions, tactical discussions and other matters. The sports education model uses the team group method to enhance teamwork and team spirit, so that the group cares, encourages, and grows with each other. With the development of the competition season, students gradually realize the importance of teamwork and no longer just focus on winning and competing alone. The arrival of the competition season makes students excited and introduces various skill exercises, tactical discussions, tactical drills, etc. in the season to preparing for the final competition. Students said that in the past, they often played alone without teamwork, but in pre-season they found that it was impossible to do so. Students gradually find that taking classes as a team can make the atmosphere more pleasant and relaxed, and can improve motivation and increase team cohesion. When the sports education model was implemented, some students were still reluctant and not very enthusiastic; even the warm-up exercises were very quiet and there was no communication. As a result, students were not motivated to learn and felt that it was a boring class. If the team group can make students realize that they are important, contributing and working in the course, it can promote the sense of belonging and cohesion, and will have a positive effect on learning.

(4) After the implementation of the sports education model, most students have obviously changed their behavior, but there are also a small number of students who have not. Some students have increased the number and time of after school exercise and actively study all information related to basketball, but a few have not changed their behavior for other reasons. Students will become active because of the competition, and will discuss and practice tactics with the team after class. Some students even change their behavior by feeling that they can help the team. Most students recognized the importance of physical fitness in pre-season and would like to improve their physical fitness; a few felt that there was no need to strengthen it. Students are affected by the COVID-19 pandemic, so those who originally wanted to practice after class have had no place to practice and discuss basketball during the holidays. No school sports venues or public spaces are open, so students can only use the short time in class or after school.

(5) The celebration activities after the game allow students to reach the final of the sports education model. They all believe that this is a valuable experience that was not available in previous physical education classes. At the beginning of the award ceremony, the students were quite excited and nervous. When they learned that they were the winners, they were very happy. Students feel that the curriculum is quite diversified, which can not only increase their knowledge but also their enjoyment of sports. The chance to finally participate in the award ceremony makes students feel that the hard work is worth it.

(6) Most students feel that the sports education model may bring different feelings when applied to other sports. Students have a positive and positive attitude toward the sports education model. This enhances emotions among peers and can also help in other learning effects.

### Problems and Solutions Encountered by Teachers When Implementing the Sports Education Model

Teachers can identify problems and solutions around several aspects, including teaching resources, student team operation, teaching content design and the impact of the COVID-19 pandemic.

From a resource perspective, there are restricted sports venues and safety issues. There are trees and soil around the schools' basketball court and, according to the schools' physical education teacher, the court has been slippery for a long time. The school intends to improve it, but it this has not happened yet. After the multi-section sports education mode, the students did not dare to sprint or make big movements. Due to the long-term wind and sand on the field, it was too slippery. In response, the researchers coordinated with other physical education teachers and booked the basketball court in advance so that the students had enough space to practice. In terms of site safety, since the school cannot immediately deal with the problem, it must be renovated during the summer vacation. Therefore, teachers will ask students to clean up the site before class to produce a safe and secure classroom environment. The researcher will watch in advance whether the teacher is using basketball in the class. If the researcher needs to use a large number of balls, he will first communicate and coordinate the number of balls with the teacher, so that the students are not waiting for too long.

Teachers experienced problems with the course content of a sports season being too standardized. There is a need to try to design the course in a step-by-step manner and consider the age and level of the students. After the lesson plan is completed, it will be implemented with the students in the sports education model. However, the researchers found that the design is too focused on action skills and the game part is lacking, which will make students feel that there is no fun in learning basketball. During the implementation of the sports education model, there was too little time for each role to practice and correct performance. This was because the pre-work warm-up exercise, the time for speaking and the time for reviewing were too long, and at the beginning, every student had to be allowed to do the exercises. Boys dominate the game more and girls feel less involved. In the sports season of the sports education model, boys and girls are more likely to practice together or discuss tactics, but when it comes to the competition season, when girls are on the court, they can often find that they are ignored or cannot get the ball. Boys think that girls have lower cognition and skills than boys, which makes them less willing to share the ball with girls during games, making girls feel that they have no place on the field. The teachers responded by increasing group games and make teaching fun. Various sports skills were learned through the process of games, so that students can first develop an interest in basketball and then continue their enthusiasm for learning. It helps to control the role practice time and explain in advance how to deal with special situations. Most of the students are experiencing being in the various roles, such as scorekeepers, timekeepers, referees etc., for the first time. The researchers set the practice time for each role and changed roles as soon as the time was up. They also explained the situations they would encounter and the solutions so as to reduce the time consumed by solving problems and let the students can practice more fluently. The researchers also changed the rules of the game to maintain at least two girls on the court during the game, allowing each team to think about how to arrange themselves in the most suitable way.

The COVID-19 pandemic also had a significant impact. The students are aware of and vigilant against the severity of the pandemic. There is no sports venue for drills and discussions after school hours. After the implementation of the sports education model, the researchers found through interviews that students would like to exercise in their spare time or on holidays, or to arrange group exercises together, in order to better perform in the final. However, they could only enjoy the short time after school and class time for drills and discussions. Before the start of each class, the researchers decided they would publicize the correct pandemic prevention methods and give students the right to wear masks in classes. They would not force students to wear masks, but would inform students that if they have heatstroke, they should immediately remove the mask for ventilation. Students were also informed that they can choose to avoid physical contact, especially during the season. The national policy is to avoid cluster infections, so sports venues in school units at all levels are not open, which means that students have no venues to exercise. The researchers provided several non-school public areas with basketball courts for students' reference, and gave each group basketball game videos to enjoy, so that although students could not exercise during the holidays, they could learn basketball through appreciation.

## Conclusions and Future Work

This study used qualitative and quantitative data to explore the impact of the implementation of the sports education model in physical education on learning motivation and learning outcomes, student's experiences of implementation and the problems and solutions encountered by teachers.

### Research Contributions

The implementation of the sports education model in basketball class has obvious positive effects on learning motivation impact, as students in the experimental group outperformed those in the traditional physical education group. There was a significant difference between the pre-test and post-test scores for the sports education model, but not for traditional physical education teaching. There were also significant differences between the models, with the sports education model being clearly superior.

The sports education model has a significant positive impact on affection, cognition and behavior, and is superior to traditional physical education. In terms of affective learning effect, the sports education model showed a significant difference between the pre- and post-test scores, while traditional physical education did not. In terms of cognitive learning effects, both models showed improvements in the learning of tactics, rules, referee signals, and movement concepts after implementation. In terms of behavioral learning effect, sports planning, sports skills, the number of after-school sports played and physical fitness all improved, while the traditional physical education teaching did not produce significant changes.

Students are highly receptive to the sports education model, have a positive attitude, improved motivation, actively change their sports behavior and effectively understand the model. Acceptance of the basketball class via the sports education model was high. The model can stimulate team cohesion and promote friendship among students through division of labor. They understand that the learning process is more important than winning or losing, can gain the experience of the competition and the enjoyment of success.

Teachers encountered a number of implementation problems but proposed solutions. Implementation and course content is constantly revised to ensure smooth teaching, which can better guide students' motivation to learn and can also enhance teachers' professional growth. The content of the teaching curriculum matches the schools' inter-class basketball game, enhances the students' athletic ability and cultivates the habit of lifelong interest in sports.

### Future Work and Limitations

This research explored the sports education model in the context of basketball in one setting. Future research are studying different sports: The research project used in this study is basketball, and it is applied to the sports education model. It is suggested that follow-up related research can explore the influence of different sports items in the sports education model on the learning effect. Choose different research objects: It is suggested that the follow-up research can select students at different stages, such as vocational, university, and different colleges, so that the research on sports education model can be more extensive and its inference can be increased.

Limitations are: The students in this study are intentionally sampled, so there may be differences due to environmental or time factors. This study uses basketball sports as a research tool, and its results should not be inferred to other sports. The ARCS Learning Motivation Scale and the Physical Education Affective Scale, which are quantitative data in this study, are that students' answering may be affected by factors such as the understanding of the question, the willingness to answer, and the authenticity of the answer, which will lead to the research results and the actual situation errors will occur.

## Data Availability Statement

The original contributions presented in the study are included in the article/Supplementary Material, further inquiries can be directed to the corresponding author.

## Author Contributions

YC, CC, and GW: conceptualization, methodology, validation, investigation, writing, funding acquisition, formal analysis, software, resources, and visualization. FS: funding acquisition, methodology, validation, writing—review and editing, and supervision. All authors contributed to the article and approved the submitted version.

## Conflict of Interest

The authors declare that the research was conducted in the absence of any commercial or financial relationships that could be construed as a potential conflict of interest.

## Publisher's Note

All claims expressed in this article are solely those of the authors and do not necessarily represent those of their affiliated organizations, or those of the publisher, the editors and the reviewers. Any product that may be evaluated in this article, or claim that may be made by its manufacturer, is not guaranteed or endorsed by the publisher.
