# Supplementary material for: The Effect of Education Model in Physical Education on Student Learning Behavior
Source: Front Psychol. 2022 Jul 8;13:944507. doi: 10.3389/fpsyg.2022.944507 (PMC9305612; doi:10.3389/fpsyg.2022.944507)
Supplement: Supplementary file 1 [file Table_1.doc]

**Appendix: The sport education model’s teaching plan table**

| Teaching stage | Section | Teaching content |
| --- | --- | --- |
| Course description  Task assignment | 1 | 1. Explain how the sports education model curriculum is implemented  2. Role assignment, group grouping, team name, team call  3. Competition draw |
| Basic movement exercises  Introduction to the competition system | 2 | 1. Announcement and explanation of the competition system  2. Each group discusses creating their own team's warm-up  3. Basic basketball movement teaching and group practice (in situ dribbling, ball feel practice) |
| Basic movement exercises  Role division and practice  Scoring, recording, referee teaching with practice | 3 | 1. Team warm-up exercises (implemented in each class)  2. Basic basketball movement teaching and group practice (walking dribble, pass in place). |
|  | 4 | 1. Basic basketball movement teaching and group practice (dribbling and comprehensive practice of passing)  2. Explain dribbling, passing fouls and referee signals indicating violations |
|  | 5 | 1. Basic basketball movement teaching and group practice (shooting)  2. Defence footwork practice, defence FAQ and discussion |
|  | 6 | 1. Basic movement review and practice  2. Scorer and recorder teaching and practice  3. Game competition (group discussion on strategy and role division) |
|  | 7 | 1. Teaching and practical training of basic offensive concepts.  2. Basketball referee signal teaching and practice  3. Game competition (group discussion on strategy and role division) |
| Role job practice (scoring, recording, refereeing)  Basic movements, general basketball rules review  Pre-match autonomy | 8 | 1. Teaching of basic walking and running concepts  2. Scorer, recorder and referee practice  3. Game competition (group discussion on strategy and role division) |
|  | 9 | 1. General review of basketball rules and basic movements  2. Scorer and referee practice |
|  | 10 | Pre-game team group tactical discussion and autonomous practice, Pre-season |
| Pre-season | 10-13 | 1. Round robin system  2. Each team leader assists team members to perform tasks  3. Apply the discussed tactics to the game  4. Discuss and revise during the competition |
| Semi-finals, Finals | 14-15 | 1. Single elimination system  2. Enjoy the game, learn to communicate and solve problems, respect opponents, sports etiquette |
| Celebration | 16 | Recognition of winners and staff |
